# Knowledge Gaps in Anesthetic Gas Utilization in a Large Academic Hospital System: A Multicenter Survey

**DOI:** 10.7759/cureus.35868

**Published:** 2023-03-07

**Authors:** Aalap C Shah, Aaron J Przybysz, Kaiyi Wang, Ian A Jones, Solmaz P Manuel, Rakhi Dayal, Michael J Jung, Nina Schlömerkemper, Seema Gandhi

**Affiliations:** 1 Anesthesiology and Perioperative Medicine, UCI (University of California Irvine) Health, Orange, USA; 2 Department of Anesthesiology, University of California Irvine (UCI), Orange, USA; 3 Office of Sustainability, University of California (UC) San Francisco Medical Center, San Francisco, USA; 4 Department of Anesthesiology and Pain Medicine, University of Washington (UW), Seattle, USA; 5 Department of Anesthesia and Perioperative Care, University of California (UC) San Francisco, San Francisco, USA; 6 Department of Anesthesia, University of California Irvine (UCI), Orange, USA; 7 Department of Anesthesiology and Pain Medicine, University of California (UC) Davis, Sacramento, USA

**Keywords:** education, sustainability, carbon dioxide emissions, volatile anesthetic, fresh gas flow

## Abstract

Inhaled anesthetics account for a significant portion of the greenhouse gases generated by perioperative services within the healthcare systems. This cross-sectional study aimed to identify knowledge gaps and practice patterns related to carbon dioxide (CO_2_) absorbents and intraoperative delivery of fresh gas flows (FGF) for future sustainability endeavors. Secondary aims focused on differences in these knowledge gaps based on the level of training. Surveys were distributed at five large academic medical centers. In addition to site-specific CO_2_ absorbent use and practice volume and experience, respondents at each institution were queried about individual practice with FGF rates during anesthetic maintenance as well as the cost-effectiveness and environmental impact of different volatile anesthetics. Results were stratified and analyzed by the level of training. In total, 368 (44% physicians, 30% residents, and 26% nurse anesthetists) respondents completed surveys. Seventy-six percent of respondents were unaware or unsure about which type of CO_2_ absorbent was in use at their hospital. Fifty-nine percent and 48% of respondents used sevoflurane and desflurane with FGF ≥1 L/min, respectively. Most participants identified desflurane as the agent with the greatest environmental impact (89.9%) and a greater proportion of anesthesiologists correctly identified isoflurane as a cost-effective anesthetic (78.3%, p=0.02). Knowledge gaps about in-use CO_2_ absorbent and optimal FGF usage were identified within the anesthesia care team. Educational initiatives to increase awareness about the carbon emissions from anesthesia and newer CO_2_ absorbents will impact the environmental and economic cost per case and align anesthesia providers toward healthcare decarbonization.

## Introduction

Inhaled volatile anesthetics, specifically halogenated methyl isopropyl ethers, such as desflurane, isoflurane, and sevoflurane, comprise the vast majority of general anesthetics administered worldwide [1]. Direct emission of volatile anesthetics contributes up to 5% of the total carbon dioxide emissions (eCO2) of the National Health Service (NHS) in the UK, more than 50% of the eCO2 from perioperative services in North America [2,3], and 0.01-0.10% of the total global eCO2 contributing to global warming [4]. Furthermore, the wasteful use of these agents contributes to increased healthcare spending without improving the quality of patient care.

In addition to eliminating or reducing the use of desflurane, the adaptation of low fresh gas flows (FGF) to decrease the consumption of sevoflurane has been proposed as a strategy to decrease emissions contributing to the greenhouse effect and ozone layer depletion [5]. However, the original recommendation by the US Food and Drug Administration (FDA) has been to avoid FGF <1 L/min and to restrict FGF of 1-2 L/min to no more than 2 minimum alveolar concentration (MAC)-hours of anesthetic delivery [6]. While there is no universal consensus, low-flow anesthesia (LFA) is most commonly defined as <1 L/min and minimal-flow anesthesia as <0.5 L/min [6]. Low FGF with sevoflurane is currently considered “off-label” by the FDA despite numerous human studies that have demonstrated the safe practice of low FGF with sevoflurane and various CO2 absorbents without any appreciable renal toxicity due to compound A [7-9]. This lack of regulatory approval is even more striking given that there are commercially available CO2 absorbents that lack strong hydroxide bases and thus do not produce compound A. For example, Amsorb, which is an absorbent developed more than 20 years ago, does not increase compound A concentration when exposed to sevoflurane (2%) in oxygen at a flow rate of 1 L/min [10].

The University of California Healthcare system has set the ambitious target of achieving carbon neutrality by 2025, galvanizing actions toward mitigating the impacts of healthcare-related emissions [11]. Inhaled anesthetics contribute to a significant portion of the Scope 1 emissions of hospitals, which are defined as emissions generated directly by sources owned and controlled by a facility [12]. In a life cycle assessment, McGain et al. demonstrated an average of 4.7 kg CO2 equivalent emission for general anesthesia with sevoflurane, versus 3.6 kg for single-use items and 2.5 kg for patient air warmer blankets [13]. Within the anesthesia department, there is a lack of data regarding the current practice patterns and knowledge gaps related to CO2 absorbents, choice of volatile anesthetics agents, and differences in maintenance FGF. The following study evaluated this knowledge gap with a multi-institution survey and better understand how these knowledge gaps differ based on the level of training.

This article was previously presented as a meeting abstract at the 2022 International Anesthesia Research Society Annual Scientific Meeting on March 18, 2022.

## Materials and methods

This survey-based study was determined to have exempt status by the University of California Institutional Review Board and the need for written informed consent was waived.

Study population

A web-based questionnaire via Qualtrics (Seattle, WA) was sent out via departmental e-mail to attending anesthesiologists, certified registered nurse anesthetists (CRNAs), and anesthesiology residents in their respective departments at five medical centers. Anonymized responses were collected during a three-month period between January 2021 and March 2021. Two reminder e-mails were sent out during this timeframe.

Survey development

The survey evaluated knowledge that the authors considered important for targeted countermeasures and aimed to assess current practices around anesthesia gas usage. The initial survey was developed via consensus from a group of six anesthesiologists with expertise and leadership roles in creating and disseminating educational content. Their decisions on what to include were based on a review of literature and knowledge in the field, current guidelines, and personal experience. Topics included knowledge about the cost-effectiveness, the environmental impact of volatile anesthetics, CO2 absorbents, and individual practice patterns. The 16-question survey was then pilot-tested by a convenience sample of 10 attending anesthesiologists and anesthesiology residents who provided critical feedback on the clarity and content of the questions via the Delphi method. Survey items were modified based on feedback to create a final survey instrument. The "prevent multiple submissions" feature in Qualtrics was activated to prevent participants from submitting multiple entries.

Statistical analysis

Descriptive statistics were used to summarize subgroups and overall scores. Categorical results are presented as counts (n) and percentages. Continuous variables are presented as mean and standard deviation for normally distributed data and median and interquartile range for non-normally distributed data. For knowledge assessment questions, responses of “unsure” and missing data were grouped with incorrect answers. For practice pattern questions, missing data were excluded when aggregated for percentage reporting and statistical significance analysis. The chi-square and Fisher's exact tests were used to analyze categorical variables and results were stratified by level of training. Statistically significant comparisons (p<0.05) were entered into a post hoc analysis to calculate residuals for cell significance. If evidence of statistically significant differences was found, a Bonferroni test was used. Data were analyzed using Software R Version 4.0.5 (R Foundation for Statistical Computing, Vienna, Austria) and Microsoft Excel Version 16.5 (Microsoft, Redmond, WA).

## Results

The survey was administered to 643 anesthesia attending, 242 CRNAs, and 304 residents among the five UC campuses. In total, 368 respondents (161 physicians (44%), 110 residents (30%), and 97 nurse anesthetists (26%) completed surveys, and the overall response rate was 30.1%. Table 1 reports the demographic characteristics of providers returning completed surveys grouped by each affiliated medical center. Seventy-six percent of all respondents were unaware or unsure about what type of CO2 absorbent they use, but there were no statistically significant differences between groups based on their level of training (Table 2) A comparatively greater proportion of anesthesiologists correctly identified isoflurane as the most cost-effective volatile anesthetic, a finding that was not statistically significant after Bonferroni correction (p=0.02). Most participants correctly identified desflurane as the least environmentally friendly volatile anesthetic, with no significant difference between groups. Response rates for each individual gas are listed in Table 1.

**Table 1:** Respondent characteristics by institution CRNA, certified registered nurse anesthetist

.	#1	#2	#3	#4	#5	Overall
	(N=89)	(N=73)	(N=37)	(N=50)	(N=119)	(N=368)
Level of Training						
Anesthesiologist	36 (40.4%)	33 (45.2%)	1 (2.7%)	20 (40.0%)	71 (59.7%)	161 (43.8%)
CRNA	33 (37.1%)	28 (38.4%)	0 (0%)	12 (24.0%)	24 (20.2%)	97 (26.4%)
Resident	20 (22.5%)	12 (16.4%)	36 (97.3%)	18 (36.0%)	24 (20.2%)	110 (29.9%)
State of Training						
Midwest	2 (2.2%)	7 (9.6%)	0 (0%)	3 (6.0%)	2 (1.7%)	14 (3.8%)
Northeast	7 (7.9%)	14 (19.2%)	0 (0%)	7 (14.0%)	19 (16.0%)	47 (12.8%)
Not specified	1 (1.1%)	2 (2.7%)	0 (0%)	1 (2.0%)	8 (6.7%)	12 (3.3%)
South/Southeast	9 (10.1%)	6 (8.2%)	1 (2.7%)	7 (14.0%)	9 (7.6%)	32 (8.7%)
West	61 (68.5%)	40 (54.8%)	36 (97.3%)	29 (58.0%)	66 (55.5%)	232 (63.0%)
Patient Population						
Single-payer system	0 (0%)	0 (0%)	0 (0%)	0 (0%)	1 (0.8%)	1 (0.3%)
Mixed: insured and uninsured	81 (91.0%)	64 (87.7%)	17 (45.9%)	41 (82.0%)	80 (67.2%)	283 (76.9%)
Mostly insured	2 (2.2%)	7 (9.6%)	20 (54.1%)	6 (12.0%)	23 (19.3%)	58 (15.8%)
Mostly uninsured/Medicaid	5 (5.6%)	2 (2.7%)	0 (0%)	0 (0%)	12 (10.1%)	19 (5.2%)
Other	0 (0%)	0 (0%)	0 (0%)	1 (2.0%)	1 (0.8%)	2 (0.5%)
Number of General Anesthetics Provided in a Week						
0-10 patients	19 (21.3%)	16 (21.9%)	12 (32.4%)	12 (24.0%)	64 (53.8%)	123 (33.4%)
11-20 patients	36 (40.4%)	35 (47.9%)	22 (59.5%)	24 (48.0%)	36 (30.3%)	153 (41.6%)
More than 20 patients	34 (38.2%)	22 (30.1%)	3 (8.1%)	14 (28.0%)	19 (16.0%)	92 (25.0%)

**Table 2:** Percentage of respondents providing the correct answer to three survey knowledge-based questions by the level of training MAC, minimum alveolar concentration

	Anesthesiologist	CRNA	Resident	Overall	P-value
Most cost-effective gas (per MAC-hour)	126 (78.3%)	66 (68.0%)	69 (62.7%)	261 (70.9%)	0.02
Least environmentally friendly gas (per MAC-hour)	149 (92.5%)	87 (89.7%)	95 (86.4%)	331 (89.9%)	0.25
Type of CO_2_ absorbent use	34 (21.1%)	32 (33.0%)	24 (21.8%)	90 (24.5%)	0.07

Fifty-nine percent and 48% of respondents used sevoflurane and desflurane with FGF ≥1 L/min, respectively (Table 3). While attending anesthesiologists reported using low FGF (<1 L/min) during sevoflurane administration more frequently, the difference between anesthesiologists, residents, and CRNAs was not statistically significant (p=0.06).

**Table 3:** Fresh gas flow (FGF) usage goal during the maintenance phase for sevoflurane and desflurane stratified by the level of training

	Anesthesiologist	CRNA	Resident	Overall	P-value
Sevoflurane					
Goal FGF < 1 L/min	73 (47.1%)	29 (31.5%)	40 (41.7%)	142 (41.4%)	0.06
Goal FGF ≥ 1 L/min	82 (52.9%)	63 (68.5%)	56 (58.3%)	201 (58.6%)	
Desflurane					
Goal FGF < 1 L/min	61 (56.0%)	32 (49.2%)	41 (49.4%)	134 (52.1%)	0.57
Goal FGF ≥ 1 L/min	48 (44.0%)	33 (50.8%)	42 (50.6%)	123 (47.9%)	

## Discussion

**Figure 1:**
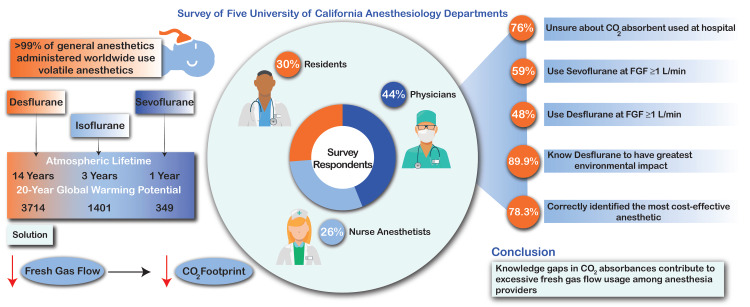
Survey of five University of California anesthesiology departments: study overview

Figure 1 provides an overview of the study methodology and conclusions. To date, very few studies have assessed knowledge gaps regarding the CO2 absorbents in use and differences in FGF administration practices. The results of this multi-institutional study reveal a deficiency in knowledge about both CO2 absorbents, leading to inefficient FGF use amongst members of the anesthesia care team. They also suggest that attending anesthesiologists are comparatively less aware of the environmental implications versus the financial implications of their anesthetic decisions. This is evidenced by the fact that attending anesthesiologists were able to distinguish themselves from others when answering questions about volatile anesthetic costs but not when answering questions about the environmental impact of their equipment or anesthetic practices. Awareness and education efforts to address these knowledge gaps are critical for the adoption of low FGF practices to reduce the environmental impact of the delivery of anesthesia, especially in the absence of evidence-based regulatory guidelines. In addition to educational initiatives, targeted interventions focused on both increasing awareness as well as optimizing intraoperative FGF delivery include clinical decision support systems and individualized and anonymized feedback including the availability of individualized reports [7,14,15].

CO2 absorbents

Significant knowledge gaps regarding CO2 absorbent used at individual institutions and intraoperative FGF delivery exist within the anesthesia care team. This study showed that most anesthesia team members do not know which CO2 absorbent they use at their home institution. Studies have demonstrated the lack of clinically significant compound A and CO production when eliminating potassium hydroxide and reducing the concentration of sodium hydroxide to <2% in CO2 absorbents [16]. As a result, a new generation of CO2 absorbents, such as lithium hydroxide-based absorbents and strong alkali-free absorbents, have been developed that contain little or no sodium hydroxide. The five affiliated medical centers have also purposefully selected non-reactive absorbents that can be used safely with low FGF to approach closed-circuit conditions and minimize anesthetic waste and emissions.

Low FGF anesthesia

Reduced FGF anesthesia, commonly defined as a flow rate between 0.5 L/min to 1 L/min, is a technique that has been safely employed to decrease carbon monoxide (CO) production, preserve humidity and body temperature, and reduce anesthetic consumption and associated costs [17,18]. In addition, both simulation and single-center prospective studies have demonstrated long-term reductions in both eCO2 and cost [14,19,20]. Volatile anesthetics undergo minimal in-vivo metabolism and are primarily (≥95%) eliminated unchanged via exhalation into waste anesthetic gases. Consequently, the environmental impacts of volatile anesthetic usage are largely dependent on the choice of gas and the FGF of its delivery. Though sevoflurane has the smallest carbon footprint of the volatile anesthetics, life cycle analyses have demonstrated that sevoflurane is the greatest contributor (and only modifiable factor) of eCO2 during general anesthesia, accounting for more than 32% of eCO2 [13]. Countries such as the United Kingdom and Germany already have recommendations in place regarding low FGF anesthesia given its efficacy in decreasing eCO2. However, in the United States, concerns regarding nephrotoxic risk based on early pre-clinical data, in combination with FDA recommendations, could be hindering the adoption of low FGF practices with sevoflurane.

Desflurane utilization

Desflurane is the least environmentally friendly volatile anesthetic, exhibiting a 10-fold greater Global Warming Potential (GWP) and a 14-fold increase in atmospheric lifetimes compared to that of sevoflurane [21-23]. A significant number of anesthesia providers today do not routinely use (or have even previously used) desflurane, as suggested by the number of respondents who were unsure about optimal maintenance FGF with this anesthetic. Desflurane initially gained traction in clinical use because of its rapid anesthetic wash-in and wash-out [24], predictable emergence in obese and morbidly obese patients, and rapid return of protective airway reflexes [25-27], which reduces the time to extubation. This anesthetic choice is particularly useful in regions where there is no post-anesthesia care unit (PACU) (i.e. Japan) and the initial recovery must happen in the operating room [28]. Subsequent studies have demonstrated that the magnitude of these clinical benefits is minimal compared to their negative environmental impact [29]. Owing to the consensus on the environmental impacts of desflurane, anesthesia care team members were able to correctly identify desflurane as the least environmental-friendly volatile anesthetic. Unlike the theoretical concerns of low FGF-associated compound A production with sevoflurane, desflurane at low FGF does not produce compound A nor does it carry the same regulatory guidelines. Despite this information, almost half of the respondents still reported targeting an FGF goal ≥ 1 L/min when using desflurane, suggesting the presence of a knowledge gap regarding CO2 absorbents that applies to all volatile anesthetics in use. Extrapolating low FGF practices to other volatile anesthetics with even greater eCO2, especially desflurane, can demonstrate a sizable reduction in GWP even for cases of minimal duration and anesthetic exposure.

Cost-effectiveness of isoflurane

It should be emphasized that this study presumes that isoflurane is more cost-effective because it costs the least in liquid form and per MAC-hour at 0.5 L/m of FGF administration [30]. However, this deduction is controversial due to concerns that isoflurane use is associated with comparatively prolonged time to extubation during cases when anesthetic duration exceeds eight hours [31]. Anesthesia costs comprise a much smaller portion of total hospital charges compared to the operating room and other facility-related fees. To this effect, Childers et al. demonstrated that the cost of operating room time across a sample of California hospitals was $37.45 in the inpatient setting and $36.14 in the ambulatory setting [32]. However, many confounders beyond the choice of volatile anesthetic affect the true cost associated with the overall length of stay, including procedure-specific considerations and postoperative recovery. Moreover, these concerns are perhaps less important in the context of the study itself, as it is unlikely that a large proportion of respondents choose an answer other than isoflurane upon consideration of the cost vs the cost-effectiveness of the gas. Taken in aggregate, the limitations discussed highlight important nuances and the challenge of perspective, be it societal, hospital, regional, or physician, when implementing clinical practice recommendations or assessing provider knowledge.

Limitations

Study limitations are primarily inherent to those of web-based surveys. Although the survey instrument was carefully developed by a team of experts, the questions did not undergo validation testing. In addition, although we aggregated data from all UC-affiliated medical centers, there were differences in the proportions of responses received from attending anesthesiologists, residents, and CRNAs at each hospital. Furthermore, practice differences with FGF between a supervising attending anesthesiologist and the resident or CRNA directly providing the anesthetic were not assessed, and thus our results might be a better indicator of provider preferences than the actual clinical practice. Next, clinician knowledge at tertiary-care academic centers in California, where this study took place and where there is significant interest in sustainability efforts, may differ from that of community practitioners or clinicians practicing in other geographic areas. It may not be appropriate to extrapolate our findings to other practice settings. Finally, we did not assess whether addressing the knowledge gap would lead to actual practice differences as part of this study.

## Conclusions

System-wide efforts are needed to address the existing knowledge gaps in CO2 absorbent properties, its relevance to low-flow anesthesia practice, and the environmental impact based on the choice of anesthetic. Anesthesiology organizations, from regional societies to national anesthesia associations, should advocate for the “off-label” use of low FGF with sevoflurane volatile anesthetic, as evidence-based practice guidelines for anesthesia professionals supersede outdated FDA guidelines. Sustainability initiatives in different perioperative departments should emphasize the contribution of anesthetic consumption to eCO2 in the context of other practices such as the use of single-use supplies including laryngoscopes and warming blankets. We recommend incorporating this valuable information into resident curricula, direct feedback, real-time clinical decision support tools, and other educational tools such as grand rounds and healthcare sustainability didactics.

## Appendices

UC-Wide Anesthesia Gases Usage Survey

Q1. How would you describe yourself and your level of training?

▢ Physician - resident

▢ Physician - attending

▢ CRNA

▢ CRNA in Training

▢ Other ________________________________________________

Q2. How do you identify yourself?

▢ Male

▢ Female

▢ Non-binary / third gender

▢ Prefer not to say

Q3. How many years have you been in practice after training?

▢ I am still in training

▢ 0-2 years

▢ 3-5 years

▢ 6-10 years

▢ 11-20 years

▢ >20 years

Q4. Where did you do your training? 

▢ USA (please specify STATE) ________________________________________________

▢ Other (please specify COUNTRY) ________________________________________________

Q5. Which institution do you practice at?

▢ UCSF Medical Center

▢ UCSD Medical Center

▢ UC Davis Medical Center

▢ UC Irvine Medical Center

▢ UCLA Medical Center

Q6. How would you describe your patient population?

▢ Mostly uninsured/Medicaid

▢ Mixed - both insured and uninsured

▢ Mostly insured

▢ 100% insured

▢ I practice in a single payer system

Q7. Approximately how many general anesthetics do you provide in a typical week?

▢ 0-5 patients

▢ 6-10 patients

▢ 11-20 patients

▢ More than 20 patients

Q8. How often do you use the following anesthetics for maintenance phase?

Desflurane

▢ 0-10% of the cases

▢ 11-25% of the cases

▢ 26-50% of the cases

▢ 51-75% of the cases

▢ 76-100% of the cases

Sevoflurane

▢ 0-10% of the cases

▢ 11-25% of the cases

▢ 26-50% of the cases

▢ 51-75% of the cases

▢ 76-100% of the cases

Isoflurane

▢ 0-10% of the cases

▢ 11-25% of the cases

▢ 26-50% of the cases

▢ 51-75% of the cases

▢ 76-100% of the cases

TIVA

▢ 0-10% of the cases

▢ 11-25% of the cases

▢ 26-50% of the cases

▢ 51-75% of the cases

▢ 76-100% of the cases

Q9. For the following types of surgical cases requiring general anesthesia, what would be your preferred primary anesthetic agent?

Desflurane

▢ Patient with renal impairment

▢ Obese patient

▢ Surgical duration < 2 hours

▢ Surgical duration >= 2 hours

Sevoflurane

▢ Patient with renal impairment

▢ Obese patient

▢ Surgical duration < 2 hours

▢ Surgical duration >= 2 hours

Isoflurane

▢ Patient with renal impairment

▢ Obese patient

▢ Surgical duration < 2 hours

▢ Surgical duration >= 2 hours

TIVA

▢ Patient with renal impairment

▢ Obese patient

▢ Surgical duration < 2 hours

▢ Surgical duration >= 2 hours

Q10. What type of CO2 absorbent do you use in your anesthesia machines? 

▢ Lithium based absorbent (Litholyme, Spiralith)

▢ Baralyme

▢ Sodasorb

▢ Medisorb

▢ Dragersorb

▢ Amsorb

▢ Yabashi Lime

▢ Soda Lime

▢ Not sure

▢ Other ________________________________________________

Q11. Do you routinely monitor end-tidal anesthetic concentrations?

▢ Yes

▢ No

▢ Do not know

Q12. Do you routinely monitor fresh gas flows?

▢ Yes

▢ No

▢ Do not know

Q13. What is your own practice regarding fresh gas flows during induction phase for most cases?

Desflurane

▢ Goal FGF <1 L/min

▢ Goal FGF 1-2 L/min

▢ Goal FGF 2-5 L/min

▢ Goal FGF 6-10 L/min

▢ Greater than 10 L/min

Sevoflurane

▢ Goal FGF <1 L/min

▢ Goal FGF 1-2 L/min

▢ Goal FGF 2-5 L/min

▢ Goal FGF 6-10 L/min

▢ Greater than 10 L/min

Isoflurane

▢ Goal FGF <1 L/min

▢ Goal FGF 1-2 L/min

▢ Goal FGF 2-5 L/min

▢ Goal FGF 6-10 L/min

▢ Greater than 10 L/min

Q14. What is your own practice regarding fresh gas flows during maintenance phase for most cases?

Desflurane

▢ Goal FGF <1 L/min

▢ Goal FGF 1-2 L/min

▢ Goal FGF 2-5 L/min

▢ Goal FGF 6-10 L/min

▢ Greater than 10 L/min

Sevoflurane

▢ Goal FGF <1 L/min

▢ Goal FGF 1-2 L/min

▢ Goal FGF 2-5 L/min

▢ Goal FGF 6-10 L/min

▢ Greater than 10 L/min

Isoflurane

▢ Goal FGF <1 L/min

▢ Goal FGF 1-2 L/min

▢ Goal FGF 2-5 L/min

▢ Goal FGF 6-10 L/min

▢ Greater than 10 L/min

Q15. In your understanding, which of the following anesthetic agent is the MOST cost-effective per MAC-hour of use?

▢ Desflurane

▢ Sevoflurane

▢ Isoflurane

Q16. In your understanding, which of the following anesthetic agent is the LEAST environmentally friendly per MAC-hour of use?

▢ Desflurane

▢ Sevoflurane

▢ Isoflurane

## References

[1] Miller AL, Theodore D, Widrich J (2021). Inhalational Anesthetic.

[2] Research GV (2019). Inhalation anesthesia market size share & trends analysis report by application (induction, maintenance), by product (sevoflurane, isoflurane, desflurane), by region, and segment forecasts, 2019 - 2025. https://www.researchandmarkets.com/reports/4621709/inhalation-anesthesia-market-size-share-and.

[3] Sherman JD, Sulbaek Andersen MP, Renwick J, McGain F (2021). Environmental sustainability in anaesthesia and critical care. Response to Br J Anaesth 2021; 126: e195-e197. Br J Anaesth.

[4] (2022). Reduce carbon footprint from inhaled anesthesia with new guidance published. https://www.asahq.org/about-asa/newsroom/news-releases/2022/06/reduce-carbon-footprint-from-inhaled-anesthesia-with-new-guidance-published.

[5] Sulbaek Andersen MP, Sander SP, Nielsen OJ, Wagner DS, Sanford TJ Jr, Wallington TJ (2010). Inhalation anaesthetics and climate change. Br J Anaesth.

[6] Mazze RI, Jamison RL (1997). Low-flow (1 l/min) sevoflurane: is it safe?. Anesthesiology.

[7] Nair BG, Peterson GN, Neradilek MB, Newman SF, Huang EY, Schwid HA (2013). Reducing wastage of inhalation anesthetics using real-time decision support to notify of excessive fresh gas flow. Anesthesiology.

[8] Kharasch ED, Frink EJ Jr, Zager R, Bowdle TA, Artru A, Nogami WM (1997). Assessment of low-flow sevoflurane and isoflurane effects on renal function using sensitive markers of tubular toxicity. Anesthesiology.

[9] Bito H, Ikeuchi Y, Ikeda K (1997). Effects of low-flow sevoflurane anesthesia on renal function: comparison with high-flow sevoflurane anesthesia and low-flow isoflurane anesthesia. Anesthesiology.

[10] Kharasch ED, Powers KM, Artru AA (2002). Comparison of Amsorb, sodalime, and Baralyme degradation of volatile anesthetics and formation of carbon monoxide and compound A in swine in vivo. Anesthesiology.

[11] NCEAS NCEAS National Center for Ecological Analysis and Synthesis. Strategic communication to achieve carbon neutrality within the University of California report of the TomKat strategic communication. https://www.nceas.ucsb.edu/tomkat-strategic-communications.

[12] Environmental Protection Agency (2022). Environmental Protection Agency. Scope 1 and scope 2 inventory guidance. https://www.epa.gov/climateleadership/scope-1-and-scope-2-inventory-guidance.

[13] McGain F, Sheridan N, Wickramarachchi K, Yates S, Chan B, McAlister S (2021). Carbon footprint of general, regional, and combined anesthesia for total knee replacements. Anesthesiology.

[14] Epstein RH, Dexter F, Maguire DP, Agarwalla NK, Gratch DM (2016). Economic and environmental considerations during low fresh gas flow volatile agent administration after change to a nonreactive carbon dioxide absorbent. Anesth Analg.

[15] Cockrell HC, Hansen EE, Gow K, Fecteau A, Greenberg SL (2023). The intersection of pediatric surgery, climate change, and equity. J Pediatr Surg.

[16] Yamakage M, Yamada S, Chen X, Iwasaki S, Tsujiguchi N, Namiki A (2000). Carbon dioxide absorbents containing potassium hydroxide produce much larger concentrations of compound A from sevoflurane in clinical practice. Anesth Analg.

[17] Baxter AD (1997). Low and minimal flow inhalational anaesthesia. Can J Anaesth.

[18] Ozcan IG, Onal O, Ozdemirkan A, Saltali A, Sari M (2022). Effects of different fresh gas flows and different anesthetics on airway temperature and humidity in surgical patients: a prospective observational study. Med Gas Res.

[19] Edmonds A, Stambaugh H, Pettey S, Daratha KB (2021). Evidence-based project: cost savings and reduction in environmental release with low-flow anesthesia. AANA J.

[20] Suttner S, Boldt J (2000). Low-flow anaesthesia. Does it have potential pharmacoeconomic consequences?. Pharmacoeconomics.

[21] Keijzer C, Perez RS, De Lange JJ (2005). Carbon monoxide production from five volatile anesthetics in dry sodalime in a patient model: halothane and sevoflurane do produce carbon monoxide; temperature is a poor predictor of carbon monoxide production. BMC Anesthesiol.

[22] Ryan SM, Nielsen CJ (2010). Global warming potential of inhaled anesthetics: application to clinical use. Anesth Analg.

[23] Sulbaek Andersen MP, Nielsen OJ, Wallington TJ, Karpichev B, Sander SP (2012). Medical intelligence article: assessing the impact on global climate from general anesthetic gases. Anesth Analg.

[24] Werner JG, Castellon-Larios K, Thongrong C, Knudsen BE, Lowery DS, Antor MA, Bergese SD (2015). Desflurane allows for a faster emergence when compared to sevoflurane without affecting the baseline cognitive recovery time. Front Med (Lausanne).

[25] Bilotta F, Doronzio A, Cuzzone V, Caramia R, Rosa G (2009). Early postoperative cognitive recovery and gas exchange patterns after balanced anesthesia with sevoflurane or desflurane in overweight and obese patients undergoing craniotomy: a prospective randomized trial. J Neurosurg Anesthesiol.

[26] La Colla L, Albertin A, La Colla G, Mangano A (2007). Faster wash-out and recovery for desflurane vs sevoflurane in morbidly obese patients when no premedication is used. Br J Anaesth.

[27] McKay RE, Malhotra A, Cakmakkaya OS, Hall KT, McKay WR, Apfel CC (2010). Effect of increased body mass index and anaesthetic duration on recovery of protective airway reflexes after sevoflurane vs desflurane. Br J Anaesth.

[28] Sugiyama D, Dexter F, Thenuwara K, Ueda K (2021). Comparison of percentage prolonged times to tracheal extubation between a Japanese teaching hospital and one in the United States, without and with a phase I postanesthesia care unit. Anesth Analg.

[29] Liu FL, Cherng YG, Chen SY (2015). Postoperative recovery after anesthesia in morbidly obese patients: a systematic review and meta-analysis of randomized controlled trials. Can J Anaesth.

[30] Moody AE, Beutler BD, Moody CE (2020). Predicting cost of inhalational anesthesia at low fresh gas flows: impact of a new generation carbon dioxide absorbent. Med Gas Res.

[31] Agoliati A, Dexter F, Lok J (2010). Meta-analysis of average and variability of time to extubation comparing isoflurane with desflurane or isoflurane with sevoflurane. Anesth Analg.

[32] Childers CP, Maggard-Gibbons M (2018). Understanding costs of care in the operating room. JAMA Surg.

